# Targeting regulatory T cells to improve vaccine immunogenicity in early life

**DOI:** 10.3389/fmicb.2014.00477

**Published:** 2014-09-11

**Authors:** Jorjoh Ndure, Katie L. Flanagan

**Affiliations:** ^1^Infant Immunology Group, Vaccinology Theme, Medical Research Council LaboratoriesFajara, The Gambia; ^2^Vaccine and Infectious Diseases Laboratory, Department of Immunology, Monash UniversityMelbourne, VIC, Australia

**Keywords:** regulatory T cells, vaccines, infants, neonates, immunogenicity, immune modulation, adjuvants

## Abstract

Human newborns and infants are bombarded with multiple pathogens on leaving the sterile intra-uterine environment, and yet have suboptimal innate immunity and limited immunological memory, thus leading to increased susceptibility to infections in early life. They are thus the target age group for a host of vaccines against common bacterial and viral pathogens. They are also the target group for many vaccines in development, including those against tuberculosis (TB), malaria, and HIV infection. However, neonatal and infant responses too many vaccines are suboptimal, and in the case of the polysaccharide vaccines, it has been necessary to develop the alternative conjugated formulations in order to induce immunity in early life. Immunoregulatory factors are an intrinsic component of natural immunity necessary to dampen or control immune responses, with the caveat that they may also decrease immunity to infections or lead to chronic infection. This review explores the key immunoregulatory factors at play in early life, with a particular emphasis on regulatory T cells (Tregs). It goes on to explore the role that Tregs play in limiting vaccine immunogenicity, and describes animal and human studies in which Tregs have been depleted in order to enhance vaccine responses. A deeper understanding of the role that Tregs play in limiting or controlling vaccine-induced immunity would provide strategies to improve vaccine immunogenicity in this critical age group. New adjuvants and drugs are being developed that can transiently suppress Treg function, and their use as part of human vaccination strategies against infections is becoming a real prospect for the future.

## INTRODUCTION

The infant immune system is uniquely adapted to meet the challenges of early life (Kollmann et al., 2012). The newborn emerges from an immune-protected environment into a world where they constantly encounter new antigens. There is therefore a need to have a series of immunoregulatory mechanisms in place in order to prevent excessive inflammation and tissue damage. At the same time the infant needs to develop immune memory upon pathogen encounter in order to be protected against future challenge. The newborn has little immunological memory, and neonates and infants are heavily reliant on innate immunity to protect them against antigenic challenge as discussed in a series of comprehensive review articles (Levy, 2007; Ghazal et al., 2013; Levy and Wynn, 2014).

In this review we discuss the regulatory factors that infants employ to suppress or control their developing immunity. We will focus on regulatory T cells (Tregs) in particular, and the potential role they play in suppressing or controlling vaccine-induced immunity in early life. We will explore the mechanisms of action used by Tregs to confer suppression, and differences in the phenotypic and functional characteristics between Tregs in infants and adults. We will discuss the role of Tregs in malaria, HIV, and hepatitis C virus (HCV) infections; and briefly describe the results of clinical trials in human infants of vaccines against these three infections. A detailed understanding of the immunoregulatory factors controlling vaccine immunogenicity in early life may provide potential strategies for improving vaccine efficacy in this vulnerable age group. We will discuss immunotherapeutic agents and vaccine adjuvants developed for use in humans that can down-modulate Treg activity and thus enhance vaccine efficacy, demonstrating that this approach is a viable option for the future.

## THE INFANT IMMUNE SYSTEM

### INNATE IMMUNITY

The innate immune system which acts as the first line of defense against infection is suboptimal at birth, and does not reach full capacity until teenage years. Innate cells express pattern recognition receptors (PRRs) which detect highly conserved pattern associate molecular patterns (PAMPs) expressed by invading pathogens or vaccines, including Toll-like receptors (TLRs) and NOD-like receptors (NLRs). Newborns and young infants have similar levels of expression of these PRRs as adults (Kollmann et al., 2012), however, responses to PRR stimulation are low at birth in part due to diminished innate signaling pathways such as IRF7 translocation (Danis et al., 2008) and TLR3 and 4 signaling (Aksoy et al., 2007). Reactivity to certain PRR agonists, e.g., TLR4 and TLR5 are acquired rapidly, and reactivity of the viral ssRNA sensing TLR7 and TLR8 receptors is robust from birth (Burl et al., 2011), hence TLR7/8 agonists are being investigated as possible adjuvants to boost immune responses to neonatal vaccines (Dowling et al., 2013). Th2 (IL-6, IL-10) and Th17 (IL-6, IL-23) polarizing cytokines dominate the innate response in early life, while TNF-α and IL-1β responses rise in the first few years of life as the former cytokines decline (Belderbos et al., 2009; Kollmann et al., 2009; Nguyen et al., 2010; Burl et al., 2011). Infant dendritic cell (DC) function is also suboptimal (De Wit et al., 2004; Goriely et al., 2004; Aksoy et al., 2007), and NK cells (Guilmot et al., 2011) and neutrophil functions (Carr, 2000) are less potent than in adults. Low complement levels in neonatal plasma are thought to increase susceptibility to certain bacterial infections, and lead to impaired adaptive immunity (Levy, 2007).

### ADAPTIVE IMMUNITY IN INFANCY

#### Infant T cell immunity

The adaptive immune system is characterized by minimal immunological memory at birth, since the newborn has been relatively protected from antigenic exposure *in utero*, and most of their T cells are of a naïve phenotype. Furthermore, high levels of TGF-β, progesterone and prostaglandin E2 *in utero* required to prevent the mother developing Th1 alloreactivity to her fetus (Philbin and Levy, 2009), alongside poor innate Th1 support (Langrish et al., 2002), result in the newborn having intrinsically skewed Th2-type immunity from birth. Additionally, the Th17 biased innate immunity in infants also results in a Th17 adaptive bias. This bias against Th1 immunity results in an increased vulnerability to microbial infections and suboptimal reactivity to many vaccines. Despite this, infants have been shown to stimulate adult level Th1 type immune responses to BCG vaccination (Marchant et al., 1999) and are thus capable of robust Th1 immunity. However, neonatal BCG vaccination results in a Th17 biased mycobacterial response compared to those receiving BCG at 4½ months of age (Burl et al., 2010), in keeping with the Th17 bias described above.

#### Infant B cell immunity

Newborn infants acquire IgG antibodies transplacentally from their mothers which provide protection against infections encountered in early life, while the other immunoglobulin subclasses are unable to cross the maternal–placenta interface. The maternally acquired antibody (MAb) levels wane over the first 6 months of life and are usually absent by 1 year of age. Several studies suggest that MAbs inhibit humoral responses to infant vaccines; including live measles vaccine (Albrecht et al., 1977) and oral poliomyelitis vaccine, and non-live vaccines including pertussis (Burstyn et al., 1983; Englund et al., 1995), tetanus and diphtheria toxoids (Bjorkholm et al., 1995), Hib conjugate vaccine (Claesson et al., 1989; Daum et al., 1991) and hepatitis A vaccine (Kanra et al., 2000); while other studies report no influence of MAbs on responses to these vaccines (Gans et al., 1998; Siegrist et al., 1998; Sallusto et al., 1999). Responses may still be protective even if MAb inhibition occurs (Jones et al., 2014), and while MAbs may interfere with the generation of humoral responses to vaccination, T cell responses do not seem to be similarly affected (Siegrist, 2003).

Human neonatal antibody responses are delayed in onset, of shorter duration, achieve lower peak levels, and have lower affinity than adults. The isotype distribution also differs, with IgG1 and IgG2 levels peaking at ∼3–4 years of age, and IgG4 only reaching adult levels at 4–6 years of age, while IgG3 is stable from birth (Ngamphaiboon et al., 1998). Histological studies of infant splenic tissue show that the marginal zone does not reach full development until 2 years of age, which alongside low complement levels (Zandvoort and Timens, 2002; Kruetzmann et al., 2003) and low expression of CD21 (complement receptor 2; Griffioen et al., 1993), would account for the delayed antibody response to T cell independent glycoproteins and polysaccharide antigens, including encapsulated bacteria such as *Streptococcus pneumoniae* and *Haemophilus influenzae*, and thus poor reactivity to polysaccharide vaccines (Adkins et al., 2004).

## IMMUNOREGULATORY FACTORS IN NEONATAL AND INFANT PLASMA

Neonatal and infant plasma contain a number of immunoregulatory factors that serve to maintain Th2 polarization, and limit pro-inflammatory innate and adaptive immunity. Newborns and infants have high levels of plasma adenosine, an endogenous purine metabolite with immunosuppressive properties. Adenosine causes mononuclear cells to produce cAMP, which acts as a second messenger to inhibit TLR-stimulated production of pro-inflammatory cytokines while polarizing toward IL-10 and Th17 cytokine production (Levy et al., 2006; Power Coombs et al., 2011; Philbin et al., 2012). Neonatal monocytes have increased sensitivity to these effects of adenosine via their adenosine A3 receptors, thus modulation of this system could potentially be used to enhance innate and therefore adaptive pro-inflammatory responses.

Several studies have shown that there are high levels of the immunosuppressive cytokine IL-10 in cord blood (CB; De Wit et al., 2004; Belderbos et al., 2009; Nguyen et al., 2010). IL-10 can be produced by most cell types of the immune system, including antigen presenting cells (APCs), granulocytes, and Th1, Th2 and many regulatory T cell subsets. IL-10 acts at a number of stages of an immune response in order to control inflammation. It inhibits the production of pro-inflammatory cytokines and chemokines by monocytes, macrophages and DCs, leading to increased IL-10 production by various T cell subsets. It suppresses both Th1 and the more recently described “Th1+Th17” cells, while enhancing CD4^+^FOXP3^+^ (forkhead box P3) regulatory T cell survival and activity, and promoting IgG and IgA class switching by B cells (Banchereau et al., 2012).

## HUMAN REGULATORY T CELL SUBTYPES AND THEIR MODES OF ACTION

Regulatory T cells are unique subpopulations of T cells that play a major role in immune homeostasis and tolerance (Sakaguchi, 2000; Belkaid et al., 2002; Mills and Mcguirk, 2004; Belkaid, 2007). Although Tregs have been shown to be beneficial in preventing an over-exuberant response and immune pathology following encounter with pathogens (Belkaid, 2008; Belkaid and Tarbell, 2009), they have also been shown to limit the favorable effector responses required for sterilizing immunity, thus allowing pathogen persistence (Kao et al., 2010).

### THYMUS DERIVED AND PERIPHERAL CD4^+^FOXP3^+^ Tregs

The Treg field was invigorated with the discovery of the transcription factor FOXP3 which is vital for the development, function and homeostasis of Tregs (Fontenot et al., 2003; Hori et al., 2003), and is thus considered the master regulator of Tregs. Its importance is further highlighted by patients with mutations in FOXP3 who develop a severe fatal disorder known as immune dysregulation, polyendocrinopathy, enteropathy, X-linked (IPEX) syndrome (Bennett and Ochs, 2001; Gambineri et al., 2003).

Recently a group of experts in the Treg field introduced a consensus nomenclature for FOXP3^+^ Tregs. They suggest replacing previously used terms which they describe as being to some extent “inaccurate and ambiguous” (Abbas et al., 2013). They recommend that the subset of FOXP3^+^ Tregs of thymic origin, which are also known as natural Tregs (nTregs), should be called thymus-derived Tregs (tTregs); while FOXP3^+^ Tregs that differentiate in the periphery should be called peripherally derived Tregs (pTreg) rather than the previous term inducible Tregs (iTregs). The pTregs are induced in the periphery in response to antigenic stimulation, and possess identical characteristics to tTregs, and therefore both subsets will be described together.

#### Phenotype of human CD4^+^FOXP3^+^ Tregs

CD4^+^FOXP3^+^ Tregs are the most widely studied Treg subset. They were first described as a subset of CD4^+^ T cells which constitutively express the interleukin 2 (IL-2) receptor alpha-chains (CD25) and can prevent the development of autoimmunity in mice (Sakaguchi et al., 1995).

Determining the precise phenotype of human CD4^+^FOXP3^+^ Tregs has proved difficult with many conflicting studies. Since CD25 is transiently expressed on conventional T cells (Hatakeyama et al., 1989), the CD25^hi^ subset is described as a more reliable marker of CD4^+^FOXP3^+^ Tregs in humans (Schmetterer et al., 2012). Human FOXP3^+^ Tregs also tend to express low levels of the IL-7 receptor CD127 (Liu et al., 2006; Seddiki et al., 2006). Therefore the most commonly analyzed phenotypes in human studies are CD4^+^CD25^+^FOXP3^+^ or CD4^+^CD25^+^CD127^lo^. Furthermore, human CD4^+^FOXP3^+^ Tregs generally express high levels of the co-inhibitory receptor cytotoxic T lymphocyte antigen 4 (CTLA4; Sansom and Walker, 2006). More recently the chemokine markers CCR4, CCR6, CXCR3, and CXCR10 have been proposed to define four distinct populations of human tTregs, each with distinct functional characteristics (Duhen et al., 2012). Each of these four Treg subsets are thought to co-localize *in vivo* with and regulate a distinct Th subset (Th1, Th2, Th17, Th22) expressing the same chemokine receptors.

CD45RA expression can be used to distinguish tTregs that are naïve or resting (rTregs; FOXP3^lo^CD45RA^+^) from the memory subset described as activated Tregs (aTregs; FOXP3^hi^CD45RA^-^; Miyara et al., 2009). The memory Tregs can be further subdivided into central memory (Treg_CM_) and effector memory (Treg_EM_) similarly to Th cells, based on the expression of chemokine receptor 7 (CCR7; Sallusto et al., 1999; Tosello et al., 2008).

Certain subpopulations among CD4^+^FOXP3^+^ Tregs are more suppressive than others. For example, Tregs expressing the tumor necrosis factor receptor 2 (TNFRII) are thought to represent a highly suppressive CD4^+^FOXP3^+^ Treg subset (Minigo et al., 2009). Those CD4^+^CD25^+^FOXP3^+^ Tregs expressing the transmembrane cyclic ADP ribose hydrolase CD38 (mainly thymic derived and in the spleen) have particularly high suppressive activity in a murine model (Patton et al., 2011). CD38 is part of a cascade involved in the production of the immunosuppressive factor adenosine from NAD^+^ (Horenstein et al., 2013) which can have immunoregulatory properties as discussed earlier. Interestingly, the majority of infant T cells express CD38 (Scalzo-Inguanti and Plebanski, 2011), and as previously mentioned infants also have high plasma levels of adenosine, but a link between these two factors has yet to be explored in neonates and infants.

#### Mechanisms of CD4^+^FOXP3^+^ Treg mediated suppression

CD4^+^FOXP3^+^ Tregs can suppress the proliferation and activation of a multitude of immune cell types including T cells, NK and NKT cells, monocytes, macrophages, B cells, DCs, and eosinophils. They employ a variety of mechanisms to mediate this suppression, and are thought to be flexible in this respect by adapting their mechanism according to their local environment (reviewed by Wing and Sakaguchi, 2012). Both IL-2 and CTLA-4-dependent mechanisms have been described, with CD25 and CTLA-4 knockout mice having a similar phenotype to Foxp3 deficient mice (Wing and Sakaguchi, 2012). It is thought that the constitutive expression of CD25 by CD4^+^FOXP3^+^ Tregs allows them to consume the available IL-2, depriving effector T cells (Teffs) and leading to effector cell death (De La Rosa et al., 2004). Those tTregs expressing CTLA-4 can suppress T cell responses via down-modulation of CD28 signaling (Walker, 2013), and reduced co-stimulatory capacity of CD80/86 expressed by DCs (Wing et al., 2011).

A commonly used mechanism of Treg action is the production of soluble inhibitory factors, including either membrane bound or released immunosuppressive cytokines IL-10, TGF-β, and IL-35 (Collison et al., 2007). FOXP3^+^ Tregs can also generate high concentrations of adenosine (Mandapathil et al., 2010) which binds to the A2a receptor on immune cells activating an immunoinhibitory loop (Sitkovsky and Ohta, 2005) which results in inhibition of T cell proliferation and cytokine production (Raskovalova et al., 2005).

### TYPE 1 REGULATORY T CELLS (Tr1)

The Tr1 Tregs are a unique Treg subset that do not rely on the expression of high levels of CD25 or FOXP3 for their function (Levings and Roncarolo, 2000). They are activated in the periphery following antigenic stimulation in the presence of IL-10 (Groux et al., 1997; Vieira et al., 2004). Recently, lymphocyte-activation gene 3 (LAG3) and CD49b have been described to represent specific markers for Tr1 cells (Gagliani et al., 2013).

The Tr1 Tregs are known to produce high levels of the immunosuppressive cytokines IL-10 and TGF-β, some IL-5, low levels of IFN-γ and IL-2, and no IL-4 (Groux et al., 1997). The secretion of IL-10 is the main mechanism by which Tr1 cells are thought to mediate suppression. The IL-10 can be either free or membrane bound, and has been shown to suppress Teff proliferation/activation both directly and indirectly via a modulation of APC function (Roncarolo et al., 2006). They have also been shown to use cell–cell contact mechanisms (Gregori et al., 2012) and the production of granzyme B and perforin (Gregori et al., 2010) to mediate suppression.

### T HELPER TYPE 3 CELLS (Th3)

This unique subset of TGF-β producing Tregs was identified in early studies investigating oral tolerance. They have been shown to suppress the proliferation and activation of Th1 cells and suppress the development of autoimmunity in the mouse model of multiple sclerosis (Chen et al., 1994). They become activated in the periphery upon encounter with a specific antigen, and suppress via the production of the inhibitory cytokine TGF-β. Some studies show that Th3 cells may have a role to play in controlling autoimmunity and allergy in humans (Andersson et al., 2002; Perez-Machado et al., 2003), but the role that this subset plays in the maintenance of immune tolerance in humans is still not clearly defined.

### CD8^+^ Tregs

While CD4^+^ Tregs have been widely studied in humans, CD8^+^ Tregs have not received the same attention. However, there is increasing evidence that subsets of CD8^+^ Tregs also play important immunoregulatory roles, and impaired CD8^+^ Treg function may lead to autoimmunity (Hu et al., 2004; Lu et al., 2008). The most widely described phenotype for CD8^+^ Tregs is CD25^+^CD28^-^ (Ciubotariu et al., 1998; Filaci et al., 2004). Other markers include CD122, CTLA-4, GITR, CD38, CD103, and CD8αα (Uss et al., 2006; Simone et al., 2008; Smith and Kumar, 2008; Liu et al., 2014); a host of different CD8 Treg subsets have been described in humans expressing various combinations of these markers (Suzuki et al., 2012). While FOXP3 expression has been described in many CD8 Treg subsets, it may also represent an activation marker rather than acting as a regulatory factor since CD8^+^FOXP3^+^ cells have been found to be minimally suppressive in some studies (Mayer et al., 2011). Mechanisms of action of CD8^+^ Tregs that have been reported include cell–cell contact mediated suppression, secretion of the suppressive cytokines IL-10 and TGF-β, and induction of APC energy (Suzuki et al., 2008). CD8^+^CD45RA^+^CCR7^+^FOXP3^+^ cells may represent a discrete subset of CD8 Tregs which interfere with the TCR signaling cascade (Suzuki et al., 2012). More extensive work is required to better understand the origin and role of CD8^+^ Tregs in immunoregulation and autoimmunity, particularly in humans.

## PHENOTYPIC AND FUNCTIONAL DIFFERENCES BETWEEN Tregs IN INFANTS COMPARED TO ADULTS

Distinct qualitative and quantitative differences have been identified between the Tregs in adults and those of infants. Most of the studies in infants have analyzed Tregs in neonatal CB for comparison with adults, and different phenotypic markers have been used to characterize the Tregs in these studies contributing to some discrepancies in the results.

FOXP3^+^ Tregs have been found in much higher levels at birth compared to adults, whether defined as CD4^+^CD25^+^CD127^lo^ (Nettenstrom et al., 2013) or CD4^+^CD25^+^FOXP3^+^ (Flanagan et al., 2010). Preterm infants have been shown to have higher levels still (Luciano et al., 2014). However, a study comparing CD4^+^CD25^+^CD127^lo^ Tregs at different age groups, showed slight increases in Treg frequencies with age: 6.10% in CB; 7.22% in adults aged 20–25 years; and 7.5% in adults over the age of 60 years (Santner-Nanan et al., 2008); and another study found that neonates had similar number of cells expressing FOXP3 compared to their mothers, and a lower number of CD4^+^CD25^bright^ cells (Ly et al., 2009). The reason for these conflicting results is not known.

Cord blood Tregs have been shown to be predominantly of the CD45RA^+^CD45RO^-^ naïve phenotype in several studies (Kanegane et al., 1991; Wing et al., 2002; Takahata et al., 2004; Ly et al., 2009; Flanagan et al., 2010). Other phenotypic differences between cord and adult Tregs include the observation that CB Tregs are mostly CD27^+^ and thus at an earlier differentiation state than their mothers; they have a lower apoptotic potential as evidenced by lower CD95/Fas expression than their mothers; and less CD62L suggesting less of a Treg_CM_ lymph node homing phenotype (Flanagan et al., 2010). CB Tregs also express less CCR6 than their matched mothers, which is the chemokine receptor that characterizes the Th17- and Th22-like Tregs (Duhen et al., 2012). Since infants are Th2 biased then their Tregs should be predominantly of a CCR4^+^CCR6^-^CXCR3^-^ Th2 Treg phenotype in keeping with the classification discussed previously (Duhen et al., 2012), although this has not been investigated in infants.

*In vitro* Treg suppression assays are difficult to perform in infants due to the lack of availability of large volumes of blood, combined with the low Treg frequencies in peripheral blood. Studies using CB are easier since large volumes are available for study. Several studies have shown that newborn CB Tregs are highly functional whereby they suppress T cell proliferation and IFN-γ production, further deviating from a Th1 response (Godfrey et al., 2005; Wing et al., 2005). Fan et al. (2012) found that CD4^+^CD25^+^ CB Tregs had a stronger immunosuppressive function than adult blood Tregs following two cycles of polyclonal stimulation. Mayer et al. (2012) found that CB CD4^+^CD25^hi^ cells failed to suppress upon TCR activation whereas those freshly purified from adult blood did, but CB Tregs became strongly suppressive after antigen-specific stimulation. Another study found that low FOXP3 expression levels by CB Tregs correlated with minimal suppressive activity, but following expansion there was a significant increase in the suppressive activity of these CB Tregs with a shift from the CD45RA^+^ to the CD45RA^-^ phenotype (Fujimaki et al., 2008). It has recently been shown that CB Tregs can be expanded more easily than adult peripheral blood Tregs, and CB Tregs are better suppressors in allogeneic mixed lymphocyte reactions than their adult counterparts (Lin et al., 2014).

Taken together these studies suggest distinct differences between infant and adult Tregs. Overall they seem to be present in higher frequencies than in adults, are more naïve and less differentiated, and are highly suppressive; all supporting an active immunoregulatory role in early life.

## THE ROLE OF Tregs IN REGULATING IMMUNITY TO MALARIA, HIV, AND HEPATITIS C VIRUS

Regulatory T cells have been implicated with an immunoregulatory role in both murine and human malaria infections (Scholzen et al., 2010). In mice, ablation of Foxp3^+^ Tregs led to increased T cell activation and decreased parasitaemia (Abel et al., 2012). *In vivo* depletion of Tregs protected mice from experimental cerebral malaria in a *Plasmodium berghei* model of infection (Wu et al., 2010). A FOXP3 promoter polymorphism in children has been associated with significant parasitaemia in a Congolese study suggesting a Treg role (Koukouikila-Koussounda et al., 2013). Malaria infected red blood cells (iRBCs) induced CD4^+^CD25^hi^FOXP3^+^ Tregs *in vitro* in healthy human volunteers (Scholzen et al., 2009). In human malaria sporozoite challenge experiments, Tregs have been shown to be induced rapidly after infection, and linked to lower pro-inflammatory cytokines and increased TGF-β production (Walther et al., 2005). Another study showed that malaria antigens can activate latent TGF-β on the surface of aTregs (Clemente et al., 2011). A study of 112 subjects in Kenya (infants to adults) found a correlation between the frequency of CD4^+^CD25^hi^ T cells and increased risk of clinical malaria, suggesting Tregs may negatively affect natural immunity to malaria in humans (Todryk et al., 2008). In naturally exposed Gambians CD4^+^FOXP3^+^CD127^lo^ Tregs during acute infection were inversely correlated with memory responses at 28 days, suggesting suppression of immune memory. In the same study, a CD4^+^FOXP3^-^CD45RO^+^ T cell population co-producing IFN-γ and IL-10 was more prevalent among children with uncomplicated malaria than those with severe disease, suggesting a beneficial immunoregulatory role for this IL-10 producing subset, presumably by limiting excessive inflammation (Walther et al., 2009). A role for the highly suppressive TNFRII^+^ Tregs in malaria parasite survival has been implicated in a study of Indonesian school children (Wammes et al., 2013). Overall, these data support an induction of Tregs during acute malaria infection which can limit the generation of immune memory and increase susceptibility to infection, but also control immunopathology and disease severity.

The role of Tregs in HIV infection remains poorly understood and the data are conflicting, in part due to the different phenotypes used to define Tregs in the various studies. However, the studies do support a regulatory role. For example, combination anti-retroviral therapy (cART) non-responders with persistent CD4 counts <200 cells/μL on therapy had higher peripheral blood Tregs and aTregs than cART responders, with higher IL-10^+^ Tregs and lower FOXP3 in lymphoid tissue (Gaardbo et al., 2014). Another longitudinal study also found higher numbers of Tregs associated with immunological non-responders defined as CD4 <500/μL (Saison et al., 2014). A study analyzing multiple Treg phenotypes in HIV infected individuals found evidence of Treg redistribution depending on HIV status (Serana et al., 2014). Untreated viraemic patients with stable CD4 counts had higher proportions of naïve Tregs with decreased Treg_CM_ compared to those on cART and healthy controls (Serana et al., 2014). The study suggests that effective cART restores Treg homeostasis since Treg subpopulations in the cART group were similar to those of healthy donors. Increased proportions and decreased numbers of Tregs associate with progression of HIV (Wang et al., 2013). Treg depletion in a murine chronic retrovirus infection model resulted in reduced viral loads (Dietze et al., 2013). In combination, the data suggest that Tregs may suppress HIV-specific immunity leading to lower CD4 counts and viral persistence.

Hepatitis C virus is characterized by its ability to establish chronic infection in the majority of those infected, and an immunoregulatory role for Tregs in this process has been well described. Chronic HCV patients have increased levels of CD4^+^ and Tr1 Tregs in peripheral blood which are thought to suppress anti-viral T cell responses leading to viral persistence (Chang, 2007). Certain HCV epitope variants have been shown to induce Tregs in HCV-infected patients (Cusick et al., 2011). Chronic HCV patients have more serum IL-10 than those with resolved infection, which is proposed to play a role in the induction of CD4^+^FOXP3^+^ Tregs in chronic HCV infection (Macdonald et al., 2002; Cusick et al., 2013); and CD49b, a marker for IL-10 producing Tr1 Treg cells, is lower in those who respond to viral therapy, thus suggesting a regulatory role for Tr1 Tregs too (Fabien et al., 2014). Indoleamine 2,3-dioxygenase (IDO) production by stimulated monocyte derived DCs was higher in HCV patients compared to healthy controls, and these DCs were more able to induce Tregs, suggesting a role for this Treg induction pathway in chronic HCV (Higashitani et al., 2013). Expression of the inhibitory signaling pathway molecule T cell immunoglobulin and mucin-domain-protein-3 (Tim-3) is upregulated on both Teff and CD4^+^CD25^+^FOXP3^+^ Tregs in chronic HCV, correlating with increased Treg and decreased Teff proliferation, suggesting that the Tim-3 pathway controls the Treg/Teff balance in chronic HCV (Moorman et al., 2012). Viral persistence following acute HCV infection is accompanied by increased plasma Galectin-9 (Gal-9) which is the ligand for Tim-3, alongside expanded Gal-9 expressing Tregs and increased expression of Tim-3 and CTLA-4 on HCV-specific CD8^+^ T cells (Kared et al., 2013). Thus high levels of Tregs likely contribute to viral persistence in HCV infection, and both FOXP3^+^ and Tr1 Tregs have been implicated. Mechanisms of Treg induction in HCV may be multifactorial but include HCV antigen driven induction, IL-10, IDO, Gal-9/Tim-3, and CTLA-4.

## ROLE OF Tregs IN CONTROLLING VACCINE IMMUNOGENICITY

The role that Tregs play in controlling or limiting vaccine immunogenicity remains to be fully determined. Given that Tregs are induced by natural infections to regulate the inflammatory response, it makes sense that Tregs would be induced as part of the immune response to vaccination, particularly for live attenuated vaccines. One might predict that their induction would play a beneficial immunoregulatory role by preventing an over-exuberant immune response to the vaccine. However, most studies suggest that Tregs can interfere with the generation of vaccine-induced immunity. Thus, depletion of Tregs pre-vaccination in murine models has been shown to enhance immune responses to some vaccines. In a DEREG mouse model, which allows for *in vivo* depletion of Foxp3^+^ Treg cells at any point during an immune response using diphtheria toxin (Lahl and Sparwasser, 2011), Treg depletion led to an enhanced anti-tumor response to vaccination against an established melanoma (Klages et al., 2010). A more recent study showed that the short term depletion of Tregs in DEREG mice greatly enhanced vaccine-induced immunity against a solid tumor; increasing NK cells and CD8 T cell activation and IFN-γ production (Mattarollo et al., 2013). Administration of vaccines with anti-CD25 monoclonal Ab has been shown to induce more durable immunity in mice compared to when the vaccine is administered alone, for both BCG and hepatitis B vaccines, which has been attributed to a depletion of CD25^+^ Tregs (Moore et al., 2005). Ho et al. (2010) showed that antigen-specific Tregs induced by environmental mycobacteria suppress Th1 immune responses, thus compromising the response to BCG vaccination in mice. They also showed a correlation between the pre-existing Tregs and the subsequent vaccine response. Murine studies of Parkinson’s disease have shown that Tregs are induced by BCG vaccination (Lacan et al., 2013).

It is difficult to translate these studies into primates and humans since murine Tregs are not phenotypically identical, and *in vivo* depletion of FOXP3^+^ Tregs in healthy humans presents logistic and ethical challenges. An oral vaccine against simian immunodeficiency virus (SIV) based on a *Lactobacillus* commensal that favors immune tolerance induction was used to induce T cell tolerance to SIV antigens in macaques (Lu et al., 2012). The vaccine-induced CD8^+^ Tregs that suppressed CD4^+^ T cell activation and *ex vivo* SIV replication, and provided sterile protection against an intrarectal SIV challenge in 15 of 16 vaccinated macaques. This strategy is thought to work because CD4^+^ T cell activation drives the initial phase of viral replication, and provides the proof-of-concept that an oral Treg inducing vaccine could prevent the establishment of HIV infection.

Using a DC-based vaccine in HIV patients undergoing cART, it was shown that depletion of the Tregs *in vitro* significantly enhanced the vaccine-induced anti-HIV-1-specific polyfunctional T cell response, suggesting that Tregs can dampen vaccine-induced immunity (Macatangay et al., 2010). This study also showed a marked increase in the CD4^+^CD25^hi^FOXP3^+^ Treg numbers following vaccination, however, this increase did not correlate with the effector CD8^+^ T cell vaccine-induced response. Increased FOXP3 mRNA expression has been demonstrated in malaria vaccinated adults; however, the authors concluded that this might be attributed to the participants being naturally exposed to the malaria parasite rather than as a result of vaccination *per se* (Mwacharo et al., 2009).

Very few studies have looked at the role that Tregs play in controlling vaccine immunogenicity in infants. Our group found no correlation between PPD-specific CD4^+^CD25^+^FOXP3^+^ Tregs or CD4^+^IL-10^+^ Tregs, or PPD stimulated total IL-10 production on the day of BCG vaccination of Gambian infants, and subsequent IFN-γ responses to PPD (Burl et al., 2010). No functional Treg assays were conducted in this study. In another study we found that placental associated malaria (PAM) infection is associated with increased malaria-specific CD4^+^CD25^+^FOXP3^+^ Tregs (Flanagan et al., 2010) and that PAM also correlates with decreased immunogenicity of BCG vaccination as evidenced by poorer PPD reactivity persisting to 1 year of age compared to PAM negative children (Walther et al., 2012). Whether the Tregs are the cause of this attenuation of BCG responses is not known.

## TARGETING Tregs *IN VIVO* TO ENHANCE VACCINE IMMUNOGENICITY

The cancer research field has made considerable advances in dissecting the role that Tregs play in cancer progression and their role in suppressing responses to cancer vaccines. Moreover, trials conducted in animal models and humans have demonstrated that certain drugs and immunotherapies can transiently decrease Treg frequencies *in vivo* leading to improved anti-tumor Teff functions, and in some cases reduced tumor load. Since Treg depletion can enhance inflammation and autoimmunity then such transient depletion, as opposed to long term effects, is desirable. In low doses, the agent cyclophosphamide transiently decreases Treg frequencies while Teff functions are preserved, leading to enhanced responses to vaccine antigens and improved vaccine immunogenicity in mouse and human cancer vaccine trials (Barbon et al., 2010; Le and Jaffee, 2012). Anti-CD25 monoclonal antibodies, which deplete Tregs *in vivo*, enhanced vaccine efficacy in mouse models of pancreatic carcinoma (Keenan et al., 2014) and melanoma (Tan et al., 2013). Basiliximab and Daclizumab are anti-human CD25 MAbs that cause both decreased number and decreased function of Tregs by blocking IL-2 signaling (Goebel et al., 2000; Kohm et al., 2006; Mitchell et al., 2011). Daclizumab has been used in several human breast cancer vaccine trials where it depleted Tregs and improved effector responses, and furthermore may reprogram naïve Tregs to become IFN-γ producers (Rech and Vonderheide, 2009; Rech et al., 2012). The human monoclonal antibody, Ipilimumab, inhibits Tregs by blocking CTLA-4; and was approved by the FDA in 2011 for use in melanoma patients (Peggs et al., 2009).

Certain innate agonists that are being used as vaccine adjuvants preferentially expand Teff over Tregs, e.g., the TLR3 agonist Poly(I:C) and the TLR9 agonist CpG-ODN; whereas others favor Treg expansion, e.g., the TLR7 agonist imiquimod (Perret et al., 2013). OX40 is part of the TNFR superfamily expressed by Teff and Tregs, and the monoclonal antibody increases Teff function while blocking Treg function. OX40 clones have been humanized as potential agents to enhance the immunogenicity of vaccines against infectious diseases (Voo et al., 2013).

An interesting approach is that of local depletion of Tregs at the site of injection of a vaccine. Chemokine receptor 4 (CCR4) antagonists can be used as vaccine adjuvants to target and decrease local recruitment of CCR4^+^ Tregs in order to amplify vaccine responses at the site of immunization (Bayry, 2014).

Therefore a number of agents that target Tregs are being used experimentally in humans in order to enhance vaccine efficacy. Some of these are non-toxic and safe for use in humans, offering the future prospect of using this approach to enhance immunogenicity of vaccines against infectious diseases including malaria, HIV, and HCV.

## TRIALS OF NOVEL VACCINES AGAINST MALARIA, HIV, AND HCV IN INFANTS

Despite the multiple obstacles to successful infant vaccination discussed above, many vaccines are currently delivered in infancy with good immunogenicity, including the live BCG, measles and yellow fever vaccines; and the inactivated diphtheria, tetanus, pertussis and hepatitis B vaccines, *H. influenzae b*, and pneumococcal conjugate vaccine. The RTS,S/AS01 malaria vaccine is the most advanced malaria vaccine in human clinical trials, having reached phase III testing in children and infants, with potential licensure in 2015. It reduced clinical and severe malaria by 56 and 47.3% respectively in children aged 5–17 months (Bejon et al., 2008; Olotu et al., 2011); but only 31.3 and 36.6% when administered in three doses with routine Expanded Program on Immunization (EPI) vaccines in the 6–12 week old age group (Rts et al., 2012). Follow up of the 5–17 month old vaccinated group over a 4 year period found protection waned to 16.8%; with waning greater among those with higher malaria exposure suggesting that natural immunity to malaria contributes to the waning (Olotu et al., 2013). Whether exposure induced Tregs play a role in this waning has not been investigated.

Fowlpox and modified vaccinia Ankara (MVA) based malaria vaccines have been tested in 1–6 year olds with no evidence of protective efficacy (Bejon et al., 2007). A blood stage vaccine FMP2.1/AS02A has been tested in Malian children aged 1–6 years with an efficacy of <10% (Laurens et al., 2013). The blood stage alum adjuvanted GMZ2 malaria vaccine elicited good inhibitory antibody levels in pre-school children (Jepsen et al., 2013). Prime-boost strategies based on chimp adenovirus vector priming followed by MVA boosting are being tested in children and infants in Africa, and while results of these trials are not yet available the adult studies have shown unprecedented immunogenicity for malaria exposed populations (Ogwang et al., 2013) and should stimulate good immunity in infants.

Only a few human HIV vaccine trials have been conducted in healthy uninfected and HIV-exposed infants. Immunogenicity was limited among healthy Gambian infants given a single dose of MVA.HIVA, but this was not surprising given that MVA alone is known to be poorly immunogenic (Afolabi et al., 2013). In a Ugandan trial, infants were vaccinated at birth, 4, 8, and 12 weeks of age with ALVAC-HIV vCP1521 (ALVAC) candidate HIV vaccine which induced low level CD4 and CD8 T cell responses at 24 months (Kaleebu et al., 2014).

The target population for HCV vaccines include intravenous drug abusers and health care professionals. However, HCV infection is common throughout the world and mother-to-child transmission is well described (Yeung et al., 2001). Thus an infant HCV vaccine would have its place, particularly in resource poor settings where the anti-viral therapies available are currently not affordable. Both therapeutic and prophylactic vaccines are being developed, and several have now entered phase I/II human clinical trials, mostly of therapeutic vaccines in chronically infected cohorts. These include recombinant protein, peptide, DNA, and vector-based vaccines aimed at producing robust anti-T cell responses (Halliday et al., 2011). As far as we are aware no HCV vaccine trials have been conducted in children.

## FUTURE PROSPECTS

There is very little in the literature regarding the role of Tregs in infants in general, and even less in respect to vaccine immunogenicity. The fact that functional Tregs are present in high numbers in infancy and have potent suppressive activity, coupled with poor immunological responses to some vaccines in this vulnerable age group, supports a need to better understand the role they play in controlling the response to childhood vaccines in particular. The data available suggest that Tregs suppress immunity to vaccines, and that they can also be induced by vaccination. We have shown that malaria, HIV, and HCV all use Tregs to evade host immune responses, therefore vaccine-induced Tregs would be predicted to reduce the protective efficacy of vaccines against these infections. A better understanding of the role that Tregs and other immunoregulatory factors play in contributing to poor vaccine immunogenicity in childhood would help with the design of better vaccines. Studies in cancer patients have shown that transient Treg inactivation or depletion is a viable approach to enhancing vaccine efficacy. A number of Treg modifying agents are available for use in humans, therefore this approach is a very real prospect for the future and may be particularly applicable to neonates and infants.

## Conflict of Interest Statement

The authors declare that the research was conducted in the absence of any commercial or financial relationships that could be construed as a potential conflict of interest.

## References

[B1] Abbas A. K. Benoist C. Bluestone J. A. Campbell D. J. Ghosh S. Hori S. (2013). Regulatory T cells: recommendations to simplify the nomenclature. *Nat. Immunol.* 14 307–308 10.1038/ni.2554 23507634

[B2] Abel S. Luckheide N. Westendorf A. M. Geffers R. Roers A. Muller W. (2012). Strong impact of CD4^+^ Foxp3^+^ regulatory T cells and limited effect of T cell-derived IL-10 on pathogen clearance during *Plasmodium yoelii* infection. *J. Immunol.* 188 5467–5477 10.4049/jimmunol.1102223 22544931

[B3] Adkins B. Leclerc C. Marshall-Clarke S. (2004). Neonatal adaptive immunity comes of age. *Nat. Rev. Immunol.* 4 553–564 10.1038/nri1394 15229474

[B4] Afolabi M. O. Ndure J. Drammeh A. Darboe F. Mehedi S. R. Rowland-Jones S. L. (2013). A phase I randomized clinical trial of candidate human immunodeficiency virus type 1 vaccine MVA.HIVA administered to Gambian infants. *PLoS ONE* 8:e78289 10.1371/journal.pone.0078289 PMC3813444 24205185

[B5] Aksoy E. Albarani V. Nguyen M. Laes J. F. Ruelle J. L. De Wit D. (2007). Interferon regulatory factor 3-dependent responses to lipopolysaccharide are selectively blunted in cord blood cells. *Blood* 109 2887–2893 10.1182/blood-2006-06-027862 17138826

[B6] Albrecht P. Ennis F. A. Saltzman E. J. Krugman S. (1977). Persistence of maternal antibody in infants beyond 12 months: mechanism of measles vaccine failure. *J. Pediatr.* 91 715–718 10.1016/S0022-3476(77)81021-4 909009

[B7] Andersson P. O. Olsson A. Wadenvik H. (2002). Reduced transforming growth factor-beta1 production by mononuclear cells from patients with active chronic idiopathic thrombocytopenic purpura. *Br. J. Haematol.* 116 862–867 10.1046/j.0007-1048.2002.03345.x 11886393

[B8] Banchereau J. Pascual V. O’Garra A. (2012). From IL-2 to IL-37: the expanding spectrum of anti-inflammatory cytokines. *Nat. Immunol.* 13 925–931 10.1038/ni.2406 22990890 PMC3609707

[B9] Barbon C. M. Yang M. Wands G. D. Ramesh R. Slusher B. S. Hedley M. L. (2010). Consecutive low doses of cyclophosphamide preferentially target Tregs and potentiate T cell responses induced by DNA PLG microparticle immunization. *Cell. Immunol.* 262 150–161 10.1016/j.cellimm.2010.02.007 20206921

[B10] Bayry J. (2014). Regulatory T cells as adjuvant target for enhancing the viral disease vaccine efficacy. *Virusdisease* 25 18–25 10.1007/s13337-013-0187-3 24426307 PMC3889236

[B11] Bejon P. Lusingu J. Olotu A. Leach A. Lievens M. Vekemans J. (2008). Efficacy of RTS,S/AS01E vaccine against malaria in children 5 to 17 months of age. *N. Engl. J. Med.* 359 2521–2532 10.1056/NEJMoa0807381 19064627 PMC2655100

[B12] Bejon P. Ogada E. Mwangi T. Milligan P. Lang T. Fegan G. (2007). Extended follow-up following a phase 2b randomized trial of the candidate malaria vaccines FP9 ME-TRAP and MVA ME-TRAP among children in Kenya. *PLoS ONE* 2:e707 10.1371/journal.pone.0000707 PMC1940326 17710125

[B13] Belderbos M. E. Van Bleek G. M. Levy O. Blanken M. O. Houben M. L. Schuijff L. (2009). Skewed pattern of Toll-like receptor 4-mediated cytokine production in human neonatal blood: low LPS-induced IL-12p70 and high IL-10 persist throughout the first month of life. *Clin. Immunol.* 133 228–237 10.1016/j.clim.2009.07.003 19648060 PMC2892115

[B14] Belkaid Y. (2007). Regulatory T cells and infection: a dangerous necessity. *Nat. Rev. Immunol.* 7 875–888 10.1038/nri2189 17948021

[B15] Belkaid Y. (2008). Paradoxical roles of Foxp3^+^ T cells during infection: from regulators to regulators. *Cell Host Microbe* 3 341–343 10.1016/j.chom.2008.05.011 18541209 PMC3415209

[B16] Belkaid Y. Piccirillo C. A. Mendez S. Shevach E. M. Sacks D. L. (2002). CD4^+^CD25^+^ regulatory T cells control *Leishmania* major persistence and immunity. *Nature* 420 502–507 10.1038/nature01152 12466842

[B17] Belkaid Y. Tarbell K. (2009). Regulatory T cells in the control of host-microorganism interactions (*). *Annu. Rev. Immunol.* 27 551–589 10.1146/annurev.immunol.021908.132723 19302048

[B18] Bennett C. L. Ochs H. D. (2001). IPEX is a unique X-linked syndrome characterized by immune dysfunction, polyendocrinopathy, enteropathy, and a variety of autoimmune phenomena. *Curr. Opin. Pediatr.* 13 533–538 10.1097/00008480-200112000-00007 11753102

[B19] Bjorkholm B. Granstrom M. Taranger J. Wahl M. Hagberg L. (1995). Influence of high titers of maternal antibody on the serologic response of infants to diphtheria vaccination at three, five and twelve months of age. *Pediatr. Infect. Dis. J.* 14 846–850 10.1097/00006454-199510000-00005 8584309

[B20] Burl S. Adetifa U. J. Cox M. Touray E. Ota M. O. Marchant A. (2010). Delaying *Bacillus* Calmette-Guerin vaccination from birth to 4 1/2 months of age reduces postvaccination Th1 and IL-17 responses but leads to comparable mycobacterial responses at 9 months of age. *J. Immunol.* 185 2620–2628 10.4049/jimmunol.1000552 20644160

[B21] Burl S. Townend J. Njie-Jobe J. Cox M. Adetifa U. J. Touray E. (2011). Age-dependent maturation of Toll-like receptor-mediated cytokine responses in Gambian infants. *PLoS ONE* 6:e18185 10.1371/journal.pone.0018185 PMC3076452 21533209

[B22] Burstyn D. G. Baraff L. J. Peppler M. S. Leake R. D. St Geme J. Jr. Manclark C. R. (1983). Serological response to filamentous hemagglutinin and lymphocytosis-promoting toxin of *Bordetella pertussis*. *Infect. Immun.* 41 1150–1156 6309662 10.1128/iai.41.3.1150-1156.1983 PMC264620

[B23] Carr R. (2000). Neutrophil production and function in newborn infants. *Br. J. Haematol.* 110 18–28 10.1046/j.1365-2141.2000.01992.x 10930976

[B24] Chang K. M. (2007). Regulatory T cells in hepatitis C virus infection. *Hepatol. Res.* 37(Suppl. 3), S327–S330 10.1111/j.1872-034X.2007.00220.x 17931182

[B25] Chen Y. Kuchroo V. K. Inobe J. Hafler D. A. Weiner H. L. (1994). Regulatory T cell clones induced by oral tolerance: suppression of autoimmune encephalomyelitis. *Science* 265 1237–1240 10.1126/science.7520605 7520605

[B26] Ciubotariu R. Colovai A. I. Pennesi G. Liu Z. Smith D. Berlocco P. (1998). Specific suppression of human CD4^+^ Th cell responses to pig MHC antigens by CD8^+^CD28^-^ regulatory T cells. *J. Immunol.* 161 5193–5202 9820490

[B27] Claesson B. A. Schneerson R. Robbins J. B. Johansson J. Lagergard T. Taranger J. (1989). Protective levels of serum antibodies stimulated in infants by two injections of *Haemophilus influenzae* type b capsular polysaccharide-tetanus toxoid conjugate. *J. Pediatr.* 114 97–100 10.1016/S0022-3476(89)80611-0 2783345

[B28] Clemente A. Caporale R. Sannella A. R. Majori G. Severini C. Fadigati G. (2011). *Plasmodium falciparum* soluble extracts potentiate the suppressive function of polyclonal T regulatory cells through activation of TGFbeta-mediated signals. *Cell. Microbiol.* 13 1328–1338 10.1111/j.1462-5822.2011.01622.x 21699642

[B29] Collison L. W. Workman C. J. Kuo T. T. Boyd K. Wang Y. Vignali K. M. (2007). The inhibitory cytokine IL-35 contributes to regulatory T-cell function. *Nature* 450 566–569 10.1038/nature06306 18033300

[B30] Cusick M. F. Libbey J. E. Cox Gill J. Fujinami R. S. Eckels D. D. (2013). CD4^+^ T-cell engagement by both wild-type and variant HCV peptides modulates the conversion of viral clearing helper T cells to Tregs. *Future Virol.* 8. 10.2217/fvl.13.49 PMC3887522 24421862

[B31] Cusick M. F. Schiller J. J. Gill J. C. Eckels D. D. (2011). Hepatitis C virus induces regulatory T cells by naturally occurring viral variants to suppress T cell responses. *Clin. Dev. Immunol.* 2011:806061 10.1155/2011/806061 PMC3004422 21197453

[B32] Danis B. George T. C. Goriely S. Dutta B. Renneson J. Gatto L. (2008). Interferon regulatory factor 7-mediated responses are defective in cord blood plasmacytoid dendritic cells. *Eur. J. Immunol.* 38 507–517 10.1002/eji.200737760 18200500

[B33] Daum R. S. Siber G. R. Ballanco G. A. Sood S. K. (1991). Serum anticapsular antibody response in the first week after immunization of adults and infants with the *Haemophilus influenzae* type b-*Neisseria meningitidis* outer membrane protein complex conjugate vaccine. *J. Infect. Dis.* 164 1154–1159 10.1093/infdis/164.6.1154 1955715

[B34] De La Rosa M. Rutz S. Dorninger H. Scheffold A. (2004). Interleukin-2 is essential for CD4^+^CD25^+^ regulatory T cell function. *Eur. J. Immunol.* 34 2480–2488 10.1002/eji.200425274 15307180

[B35] De Wit D. Olislagers V. Goriely S. Vermeulen F. Wagner H. Goldman M. (2004). Blood plasmacytoid dendritic cell responses to CpG oligodeoxynucleotides are impaired in human newborns. *Blood* 103 1030–1032 10.1182/blood-2003-04-1216 14504106

[B36] Dietze K. K. Zelinskyy G. Liu J. Kretzmer F. Schimmer S. Dittmer U. (2013). Combining regulatory T cell depletion and inhibitory receptor blockade improves reactivation of exhausted virus-specific CD8^+^ T cells and efficiently reduces chronic retroviral loads. *PLoS Pathog.* 9:e1003798 10.1371/journal.ppat.1003798 PMC3855586 24339778

[B37] Dowling D. J. Tan Z. Prokopowicz Z. M. Palmer C. D. Matthews M. A. Dietsch G. N. (2013). The ultra-potent and selective TLR8 agonist VTX-294 activates human newborn and adult leukocytes. *PLoS ONE* 8:e58164 10.1371/journal.pone.0058164 PMC3587566 23483986

[B38] Duhen T. Duhen R. Lanzavecchia A. Sallusto F. Campbell D. J. (2012). Functionally distinct subsets of human FOXP3^+^ Treg cells that phenotypically mirror effector Th cells. *Blood* 119 4430–4440 10.1182/blood-2011-11-392324 22438251 PMC3362361

[B39] Englund J. A. Anderson E. L. Reed G. F. Decker M. D. Edwards K. M. Pichichero M. E. (1995). The effect of maternal antibody on the serologic response and the incidence of adverse reactions after primary immunization with acellular and whole-cell pertussis vaccines combined with diphtheria and tetanus toxoids. *Pediatrics* 96 580–584 7659480

[B40] Fabien S. Olivier M. Khaldoun G. Vivian V. Lynda A. Laurissa O. (2014). CD49b, a major marker of regulatory T-cells type 1, predicts the response to antiviral therapy of recurrent hepatitis C after liver transplantation. *Biomed Res. Int.* 2014:290878 10.1155/2014/290878 PMC3915765 24575405

[B41] Fan H. Yang J. Hao J. Ren Y. Chen L. Li G. (2012). Comparative study of regulatory T cells expanded ex vivo from cord blood and adult peripheral blood. *Immunology* 136 218–230 10.1111/j.1365-2567.2012.03573.x 22348606 PMC3403260

[B42] Filaci G. Fravega M. Negrini S. Procopio F. Fenoglio D. Rizzi M. (2004). Nonantigen specific CD8^+^ T suppressor lymphocytes originate from CD8^+^CD28^-^ T cells and inhibit both T-cell proliferation and CTL function. *Hum. Immunol.* 65 142–156 10.1016/j.humimm.2003.12.001 14969769

[B43] Flanagan K. L. Halliday A. Burl S. Landgraf K. Jagne Y. J. Noho-Konteh F. (2010). The effect of placental malaria infection on cord blood and maternal immunoregulatory responses at birth. *Eur. J. Immunol.* 40 1062–1072 10.1002/eji.200939638 20039298

[B44] Fontenot J. D. Gavin M. A. Rudensky A. Y. (2003). Foxp3 programs the development and function of CD4^+^CD25^+^ regulatory T cells. *Nat. Immunol.* 4 330–336 10.1038/ni904 12612578

[B45] Fujimaki W. Takahashi N. Ohnuma K. Nagatsu M. Kurosawa H. Yoshida S. (2008). Comparative study of regulatory T cell function of human CD25CD4 T cells from thymocytes, cord blood, and adult peripheral blood. *Clin. Dev. Immunol.* 2008:305859 10.1155/2008/305859 PMC2547481 18815628

[B46] Gaardbo J. C. Hartling H. J. Ronit A. Springborg K. Gjerdrum L. M. Ralfkiaer E. (2014). Regulatory T cells in HIV-infected immunological non-responders are increased in blood but depleted in lymphoid tissue and predict immunological reconstitution. *J. Acquir. Immune Defic. Syndr.* 66 349–357 10.1097/QAI.0000000000000173 24784764

[B47] Gagliani N. Magnani C. F. Huber S. Gianolini M. E. Pala M. Licona-Limon P. (2013). Coexpression of CD49b and LAG-3 identifies human and mouse T regulatory type 1 cells. *Nat. Med.* 19 739–746 10.1038/nm.3179 23624599

[B48] Gambineri E. Torgerson T. R. Ochs H. D. (2003). Immune dysregulation, polyendocrinopathy, enteropathy, and X-linked inheritance (IPEX), a syndrome of systemic autoimmunity caused by mutations of FOXP3, a critical regulator of T-cell homeostasis. *Curr. Opin. Rheumatol.* 15 430–435 10.1097/00002281-200307000-00010 12819471

[B49] Gans H. A. Arvin A. M. Galinus J. Logan L. Dehovitz R. Maldonado Y. (1998). Deficiency of the humoral immune response to measles vaccine in infants immunized at age 6 months. *JAMA* 280 527–532 10.1001/jama.280.6.527 9707142

[B50] Ghazal P. Dickinson P. Smith C. L. (2013). Early life response to infection. *Curr. Opin. Infect. Dis.* 26 213–218 10.1097/QCO.0b013e32835fb8bf 23449137

[B51] Godfrey W. R. Spoden D. J. Ge Y. G. Baker S. R. Liu B. Levine B. L. (2005). Cord blood CD4(+)CD25(+)-derived T regulatory cell lines express FoxP3 protein and manifest potent suppressor function. *Blood* 105 750–758 10.1182/blood-2004-06-2467 15374887

[B52] Goebel J. Stevens E. Forrest K. Roszman T. L. (2000). Daclizumab (Zenapax) inhibits early interleukin-2 receptor signal transduction events. *Transpl. Immunol.* 8 153–159 10.1016/S0966-3274(00)00021-6 11147695

[B53] Goriely S. Van Lint C. Dadkhah R. Libin M. De Wit D. Demonte D. (2004). A defect in nucleosome remodeling prevents IL-12(p35) gene transcription in neonatal dendritic cells. *J. Exp. Med.* 199 1011–1016 10.1084/jem.20031272 15051764 PMC2211877

[B54] Gregori S. Goudy K. S. Roncarolo M. G. (2012). The cellular and molecular mechanisms of immuno-suppression by human type 1 regulatory T cells. *Front. Immunol.* 3:30 10.3389/fimmu.2012.00030 PMC3342353 22566914

[B55] Gregori S. Tomasoni D. Pacciani V. Scirpoli M. Battaglia M. Magnani C. F. (2010). Differentiation of type 1 T regulatory cells (Tr1) by tolerogenic DC-10 requires the IL-10-dependent ILT4/HLA-G pathway. *Blood* 116 935–944 10.1182/blood-2009-07-234872 20448110

[B56] Griffioen A. W. Franklin S. W. Zegers B. J. Rijkers G. T. (1993). Expression and functional characteristics of the complement receptor type 2 on adult and neonatal B lymphocytes. *Clin. Immunol. Immunopathol.* 69 1–8 10.1006/clin.1993.1142 8403536

[B57] Groux H. O’Garra A. Bigler M. Rouleau M. Antonenko S. De Vries J. E. (1997). A CD4^+^ T-cell subset inhibits antigen-specific T-cell responses and prevents colitis. *Nature* 389 737–742 10.1038/39614 9338786

[B58] Guilmot A. Hermann E. Braud V. M. Carlier Y. Truyens C. (2011). Natural killer cell responses to infections in early life. *J. Innate Immun.* 3 280–288 10.1159/000323934 21411972

[B59] Halliday J. Klenerman P. Barnes E. (2011). Vaccination for hepatitis C virus: closing in on an evasive target. *Expert Rev. Vaccines* 10 659–672 10.1586/erv.11.55 21604986 PMC3112461

[B60] Hatakeyama M. Tsudo M. Minamoto S. Kono T. Doi T. Miyata T. (1989). Interleukin-2 receptor beta chain gene: generation of three receptor forms by cloned human alpha and beta chain cDNA’s. *Science* 244 551–556 10.1126/science.2785715 2785715

[B61] Higashitani K. Kanto T. Kuroda S. Yoshio S. Matsubara T. Kakita N. (2013). Association of enhanced activity of indoleamine 2,3-dioxygenase in dendritic cells with the induction of regulatory T cells in chronic hepatitis C infection. *J. Gastroenterol.* 48 660–670 10.1007/s00535-012-0667-z 22976933

[B62] Ho P. Wei X. Seah G. T. (2010). Regulatory T cells induced by *Mycobacterium chelonae* sensitization influence murine responses to bacille Calmette-Guerin. *J. Leukoc. Biol.* 88 1073–1080 10.1189/jlb.0809582 20651297

[B63] Horenstein A. L. Chillemi A. Zaccarello G. Bruzzone S. Quarona V. Zito A. (2013). A CD38/CD203a/CD73 ectoenzymatic pathway independent of CD39 drives a novel adenosinergic loop in human T lymphocytes. *Oncoimmunology* 2:e26246 10.4161/onci.26246 PMC3850273 24319640

[B64] Hori S. Nomura T. Sakaguchi S. (2003). Control of regulatory T cell development by the transcription factor Foxp3. *Science* 299 1057–1061 10.1126/science.1079490 12522256

[B65] Hu D. Ikizawa K. Lu L. Sanchirico M. E. Shinohara M. L. Cantor H. (2004). Analysis of regulatory CD8 T cells in Qa-1-deficient mice. *Nat. Immunol.* 5 516–523 10.1038/ni1063 15098030

[B66] Jepsen M. P. Jogdand P. S. Singh S. K. Esen M. Christiansen M. Issifou S. (2013). The malaria vaccine candidate GMZ2 elicits functional antibodies in individuals from malaria endemic and non-endemic areas. *J. Infect. Dis.* 208 479–488 10.1093/infdis/jit185 23624363

[B67] Jones C. Pollock L. Barnett S. M. Battersby A. Kampmann B. (2014). The relationship between concentration of specific antibody at birth and subsequent response to primary immunization. *Vaccine* 32 996–1002 10.1016/j.vaccine.2013.11.104 24342250

[B68] Kaleebu P. Njai H. F. Wang L. Jones N. Ssewanyana I. Richardson P. (2014). Immunogenicity of ALVAC-HIV vCP1521 in infants of HIV-1-infected women in Uganda (HPTN 027): the first pediatric HIV vaccine trial in Africa. *J. Acquir. Immune Defic. Syndr.* 65 268–277 10.1097/01.qai.0000435600.65845.31 24091694 PMC4171956

[B69] Kanegane H. Miyawaki T. Kato K. Yokoi T. Uehara T. Yachie A. (1991). A novel subpopulation of CD45RA^+^ CD4^+^ T cells expressing IL-2 receptor alpha-chain (CD25) and having a functionally transitional nature into memory cells. *Int. Immunol.* 3 1349–1356 10.1093/intimm/3.12.1349 1685671

[B70] Kanra G. Yalcin S. S. Ceyhan M. Yurdakok K. (2000). Clinical trial to evaluate immunogenicity and safety of inactivated hepatitis A vaccination starting at 2-month-old children. *Turk. J. Pediatr.* 42 105–108 10936974

[B71] Kao J. Y. Zhang M. Miller M. J. Mills J. C. Wang B. Liu M. (2010). *Helicobacter pylori* immune escape is mediated by dendritic cell-induced Treg skewing and Th17 suppression in mice. *Gastroenterology* 138 1046–1054 10.1053/j.gastro.2009.11.043 19931266 PMC2831148

[B72] Kared H. Fabre T. Bedard N. Bruneau J. Shoukry N. H. (2013). Galectin-9 and IL-21 mediate cross-regulation between Th17 and Treg cells during acute hepatitis C. *PLoS Pathog.* 9:e1003422 10.1371/journal.ppat.1003422 PMC3688567 23818845

[B73] Keenan B. P. Saenger Y. Kafrouni M. I. Leubner A. Lauer P. Maitra A. (2014). A *Listeria* vaccine and depletion of T-regulatory cells activate immunity against early stage pancreatic intraepithelial neoplasms and prolong survival of mice. *Gastroenterology* 146 1784 e6–1794e6. 10.1053/j.gastro.2014.02.055 24607504 PMC4035450

[B74] Klages K. Mayer C. T. Lahl K. Loddenkemper C. Teng M. W. Ngiow S. F. (2010). Selective depletion of Foxp3^+^ regulatory T cells improves effective therapeutic vaccination against established melanoma. *Cancer Res.* 70 7788–7799 10.1158/0008-5472.CAN-10-1736 20924102

[B75] Kohm A. P. Mcmahon J. S. Podojil J. R. Begolka W. S. Degutes M. Kasprowicz D. J. (2006). Cutting Edge: Anti-CD25 monoclonal antibody injection results in the functional inactivation, not depletion, of CD4^+^CD25^+^ T regulatory cells. *J. Immunol.* 176 3301–3305 10.4049/jimmunol.176.6.3301 16517695

[B76] Kollmann T. R. Crabtree J. Rein-Weston A. Blimkie D. Thommai F. Wang X. Y. (2009). Neonatal innate TLR-mediated responses are distinct from those of adults. *J. Immunol.* 183 7150–7160 10.4049/jimmunol.0901481 19917677 PMC4556237

[B77] Kollmann T. R. Levy O. Montgomery R. R. Goriely S. (2012). Innate immune function by Toll-like receptors: distinct responses in newborns and the elderly. *Immunity* 37 771–783 10.1016/j.immuni.2012.10.014 23159225 PMC3538030

[B78] Koukouikila-Koussounda F. Ntoumi F. Ndounga M. Tong H. V. Abena A. A. Velavan T. P. (2013). Genetic evidence of regulatory gene variants of the STAT6, IL10R and FOXP3 locus as a susceptibility factor in uncomplicated malaria and parasitaemia in Congolese children. *Malar. J.* 12:9 10.1186/1475-2875-12-9 PMC3547756 23297791

[B79] Kruetzmann S. Rosado M. M. Weber H. Germing U. Tournilhac O. Peter H. H. (2003). Human immunoglobulin M memory B cells controlling *Streptococcus pneumoniae* infections are generated in the spleen. *J. Exp. Med.* 197 939–945 10.1084/jem.20022020 12682112 PMC2193885

[B80] Lacan G. Dang H. Middleton B. Horwitz M. A. Tian J. Melega W. P. (2013). *Bacillus* Calmette-Guerin vaccine-mediated neuroprotection is associated with regulatory T-cell induction in the 1-methyl-4-phenyl-1,2,3,6-tetrahydropyridine mouse model of Parkinson’s disease. *J. Neurosci. Res.* 91 1292–1302 10.1002/jnr.23253 23907992 PMC5800426

[B81] Lahl K. Sparwasser T. (2011). In vivo depletion of FoxP3^+^ Tregs using the DEREG mouse model. *Methods Mol. Biol.* 707 157–172 10.1007/978-1-61737-979-6_10 21287334

[B82] Langrish C. L. Buddle J. C. Thrasher A. J. Goldblatt D. (2002). Neonatal dendritic cells are intrinsically biased against Th-1 immune responses. *Clin. Exp. Immunol.* 128 118–123 10.1046/j.1365-2249.2002.01817.x 11982599 PMC1906380

[B83] Laurens M. B. Thera M. A. Coulibaly D. Ouattara A. Kone A. K. Guindo A. B. (2013). Extended safety, immunogenicity and efficacy of a blood-stage malaria vaccine in Malian children: 24-month follow-up of a randomized, double-blinded phase 2 trial. *PLoS ONE* 8:e79323 10.1371/journal.pone.0079323 PMC3832522 24260195

[B84] Le D. T. Jaffee E. M. (2012). Regulatory T-cell modulation using cyclophosphamide in vaccine approaches: a current perspective. *Cancer Res.* 72 3439–3444 10.1158/0008-5472.CAN-11-3912 22761338 PMC3399042

[B85] Levings M. K. Roncarolo M. G. (2000). T-regulatory 1 cells: a novel subset of CD4 T cells with immunoregulatory properties. *J. Allergy Clin. Immunol.* 106 S109–S112 10.1067/mai.2000.106635 10887343

[B86] Levy O. (2007). Innate immunity of the newborn: basic mechanisms and clinical correlates. *Nat. Rev. Immunol.* 7 379–390 10.1038/nri2075 17457344

[B87] Levy O. Coughlin M. Cronstein B. N. Roy R. M. Desai A. Wessels M. R. (2006). The adenosine system selectively inhibits TLR-mediated TNF-alpha production in the human newborn. *J. Immunol.* 177 1956–1966 10.4049/jimmunol.177.3.1956 16849509 PMC2881468

[B88] Levy O. Wynn J. L. (2014). A prime time for trained immunity: innate immune memory in newborns and infants. *Neonatology* 105 136–141 10.1159/000356035 24356292 PMC3946366

[B89] Lin S. J. Lu C. H. Yan D. C. Lee P. T. Hsiao H. S. Kuo M. L. (2014). Expansion of regulatory T cells from umbilical cord blood and adult peripheral blood CD4CD25 T cells. *Immunol. Res.* 10.1007/s12026-014-8488-1 [Epub ahead of print] 24515612

[B90] Liu W. Putnam A. L. Xu-Yu Z. Szot G. L. Lee M. R. Zhu S. (2006). CD127 expression inversely correlates with FoxP3 and suppressive function of human CD4^+^ T reg cells. *J. Exp. Med.* 203 1701–1711 10.1084/jem.20060772 16818678 PMC2118339

[B91] Liu Y. Lan Q. Lu L. Chen M. Xia Z. Ma J. (2014). Phenotypic and functional characteristic of a newly identified CD8^+^ Foxp3^-^ CD103^+^ regulatory T cells. *J. Mol. Cell Biol.* 6 81–92 10.1093/jmcb/mjt026 23861553 PMC3927769

[B92] Lu L. Kim H. J. Werneck M. B. Cantor H. (2008). Regulation of CD8^+^ regulatory T cells: Interruption of the NKG2A-Qa-1 interaction allows robust suppressive activity and resolution of autoimmune disease. *Proc. Natl. Acad. Sci. U.S.A.* 105 19420–19425 10.1073/pnas.0810383105 19047627 PMC2614776

[B93] Lu W. Chen S. Lai C. Guo W. Fu L. Andrieu J. M. (2012). Induction of CD8^+^ regulatory T cells protects macaques against SIV challenge. *Cell Rep.* 2 1736–1746 10.1016/j.celrep.2012.11.016 23260669

[B94] Luciano A. A. Arbona-Ramirez I. M. Ruiz R. Llorens-Bonilla B. J. Martinez-Lopez D. G. Funderburg N. (2014). Alterations in regulatory T cell subpopulations seen in preterm infants. *PLoS ONE* 9:e95867 10.1371/journal.pone.0095867 PMC4010410 24796788

[B95] Ly N. P. Ruiz-Perez B. Mcloughlin R. M. Visness C. M. Wallace P. K. Cruikshank W. W. (2009). Characterization of regulatory T cells in urban newborns. *Clin. Mol. Allergy* 7:8 10.1186/1476-7961-7-8 PMC2717905 19586545

[B96] Macatangay B. J. Szajnik M. E. Whiteside T. L. Riddler S. A. Rinaldo C. R. (2010). Regulatory T cell suppression of Gag-specific CD8 T cell polyfunctional response after therapeutic vaccination of HIV-1-infected patients on ART. *PLoS ONE* 5:e9852 10.1371/journal.pone.0009852 PMC2844424 20352042

[B97] Macdonald A. J. Duffy M. Brady M. T. Mckiernan S. Hall W. Hegarty J. (2002). CD4 T helper type 1 and regulatory T cells induced against the same epitopes on the core protein in hepatitis C virus-infected persons. *J. Infect. Dis.* 185 720–727 10.1086/339340 11920289

[B98] Mandapathil M. Hilldorfer B. Szczepanski M. J. Czystowska M. Szajnik M. Ren J. (2010). Generation and accumulation of immunosuppressive adenosine by human CD4^+^CD25highFOXP3^+^ regulatory T cells. *J. Biol. Chem.* 285 7176–7186 10.1074/jbc.M109.047423 19858205 PMC2844167

[B99] Marchant A. Goetghebuer T. Ota M. O. Wolfe I. Ceesay S. J. De Groote D. (1999). Newborns develop a Th1-type immune response to *Mycobacterium bovis Bacillus* Calmette-Guerin vaccination. *J. Immunol.* 163 2249–2255 10438968

[B100] Mattarollo S. R. Steegh K. Li M. Duret H. Foong Ngiow S. Smyth M. J. (2013). Transient Foxp3(+) regulatory T-cell depletion enhances therapeutic anticancer vaccination targeting the immune-stimulatory properties of NKT cells. *Immunol. Cell Biol.* 91 105–114 10.1038/icb.2012.58 23090488

[B101] Mayer C. T. Floess S. Baru A. M. Lahl K. Huehn J. Sparwasser T. (2011). CD8^+^ Foxp3^+^ T cells share developmental and phenotypic features with classical CD4^+^ Foxp3^+^ regulatory T cells but lack potent suppressive activity. *Eur. J. Immunol.* 41 716–725 10.1002/eji.201040913 21312192

[B102] Mayer E. Bannert C. Gruber S. Klunker S. Spittler A. Akdis C. A. (2012). Cord blood derived CD4^+^ CD25(high) T cells become functional regulatory T cells upon antigen encounter. *PLoS ONE* 7:e29355 10.1371/journal.pone.0029355 PMC3260151 22272233

[B103] Mills K. H. Mcguirk P. (2004). Antigen-specific regulatory T cells – their induction and role in infection. *Semin. Immunol.* 16 107–117 10.1016/j.smim.2003.12.006 15036234

[B104] Minigo G. Woodberry T. Piera K. A. Salwati E. Tjitra E. Kenangalem E. (2009). Parasite-dependent expansion of TNF receptor II-positive regulatory T cells with enhanced suppressive activity in adults with severe malaria. *PLoS Pathog.* 5:e1000402 10.1371/journal.ppat.1000402 PMC2668192 19390618

[B105] Mitchell D. A. Cui X. Schmittling R. J. Sanchez-Perez L. Snyder D. J. Congdon K. L. (2011). Monoclonal antibody blockade of IL-2 receptor alpha during lymphopenia selectively depletes regulatory T cells in mice and humans. *Blood* 118 3003–3012 10.1182/blood-2011-02-334565 21768296 PMC3175779

[B106] Miyara M. Yoshioka Y. Kitoh A. Shima T. Wing K. Niwa A. (2009). Functional delineation and differentiation dynamics of human CD4^+^ T cells expressing the FoxP3 transcription factor. *Immunity* 30 899–911 10.1016/j.immuni.2009.03.019 19464196

[B107] Moore A. C. Gallimore A. Draper S. J. Watkins K. R. Gilbert S. C. Hill A. V. (2005). Anti-CD25 antibody enhancement of vaccine-induced immunogenicity: increased durable cellular immunity with reduced immunodominance. *J. Immunol.* 175 7264–7273 10.4049/jimmunol.175.11.7264 16301631

[B108] Moorman J. P. Wang J. M. Zhang Y. Ji X. J. Ma C. J. Wu X. Y. (2012). Tim-3 pathway controls regulatory and effector T cell balance during hepatitis C virus infection. *J. Immunol.* 189 755–766 10.4049/jimmunol.1200162 22706088 PMC3392408

[B109] Mwacharo J. Dunachie S. J. Kai O. Hill A. V. Bejon P. Fletcher H. A. (2009). Quantitative PCR evaluation of cellular immune responses in Kenyan children vaccinated with a candidate malaria vaccine. *PLoS ONE* 4:e8434 10.1371/journal.pone.0008434 PMC2792766 20037644

[B110] Nettenstrom L. Alderson K. Raschke E. E. Evans M. D. Sondel P. M. Olek S. (2013). An optimized multi-parameter flow cytometry protocol for human T regulatory cell analysis on fresh and viably frozen cells, correlation with epigenetic analysis, and comparison of cord and adult blood. *J. Immunol. Methods* 387 81–88 10.1016/j.jim.2012.09.014 23058673 PMC3529842

[B111] Ngamphaiboon J. Theamboonlert A. Poovorawan Y. (1998). Serum IgG subclass levels in a group of healthy Thai children. *Asian Pac. J. Allergy Immunol.* 16 49–55 9681129

[B112] Nguyen M. Leuridan E. Zhang T. De Wit D. Willems F. Van Damme P. (2010). Acquisition of adult-like TLR4 and TLR9 responses during the first year of life. *PLoS ONE* 5:e10407 10.1371/journal.pone.0010407 PMC2861003 20442853

[B113] Ogwang C. Afolabi M. Kimani D. Jagne Y. J. Sheehy S. H. Bliss C. M. (2013). Safety and immunogenicity of heterologous prime-boost immunisation with *Plasmodium falciparum* malaria candidate vaccines, ChAd63 ME-TRAP and MVA ME-TRAP, in healthy Gambian and Kenyan adults. *PLoS ONE* 8:e57726 10.1371/journal.pone.0057726 PMC3602521 23526949

[B114] Olotu A. Fegan G. Wambua J. Nyangweso G. Awuondo K. O. Leach A. (2013). Four-year efficacy of RTS,S/AS01E and its interaction with malaria exposure. *N. Engl. J. Med.* 368 1111–1120 10.1056/NEJMoa1207564 23514288 PMC5156295

[B115] Olotu A. Lusingu J. Leach A. Lievens M. Vekemans J. Msham S. (2011). Efficacy of RTS,S/AS01E malaria vaccine and exploratory analysis on anti-circumsporozoite antibody titres and protection in children aged 5-17 months in Kenya and Tanzania: a randomised controlled trial. *Lancet Infect. Dis.* 11 102–109 10.1016/S1473-3099(10)70262-0 21237715 PMC3341451

[B116] Patton D. T. Wilson M. D. Rowan W. C. Soond D. R. Okkenhaug K. (2011). The PI3K p110delta regulates expression of CD38 on regulatory T cells. *PLoS ONE* 6:e17359 10.1371/journal.pone.0017359 PMC3046981 21390257

[B117] Peggs K. S. Quezada S. A. Chambers C. A. Korman A. J. Allison J. P. (2009). Blockade of CTLA-4 on both effector and regulatory T cell compartments contributes to the antitumor activity of anti-CTLA-4 antibodies. *J. Exp. Med.* 206 1717–1725 10.1084/jem.20082492 19581407 PMC2722174

[B118] Perez-Machado M. A. Ashwood P. Thomson M. A. Latcham F. Sim R. Walker-Smith J. A. (2003). Reduced transforming growth factor-beta1-producing T cells in the duodenal mucosa of children with food allergy. *Eur. J. Immunol.* 33 2307–2315 10.1002/eji.200323308 12884306

[B119] Perret R. Sierro S. R. Botelho N. K. Corgnac S. Donda A. Romero P. (2013). Adjuvants that improve the ratio of antigen-specific effector to regulatory T cells enhance tumor immunity. *Cancer Res.* 73 6597–6608 10.1158/0008-5472.CAN-13-0875 24048821

[B120] Philbin V. J. Dowling D. J. Gallington L. C. Cortes G. Tan Z. Suter E. E. (2012). Imidazoquinoline Toll-like receptor 8 agonists activate human newborn monocytes and dendritic cells through adenosine-refractory and caspase-1-dependent pathways. *J. Allergy Clin. Immunol.* 130 195 e9–204e9. 10.1016/j.jaci.2012.02.042 22521247 PMC3387351

[B121] Philbin V. J. Levy O. (2009). Developmental biology of the innate immune response: implications for neonatal and infant vaccine development. *Pediatr. Res.* 65 98R–105R 10.1203/PDR.0b013e31819f195d PMC2795575 19918215

[B122] Power Coombs M. R. Belderbos M. E. Gallington L. C. Bont L. Levy O. (2011). Adenosine modulates Toll-like receptor function: basic mechanisms and translational opportunities. *Expert Rev. Anti Infect. Ther.* 9 261–269 10.1586/eri.10.158 21342073 PMC4052217

[B123] Raskovalova T. Huang X. Sitkovsky M. Zacharia L. C. Jackson E. K. Gorelik E. (2005). Gs protein-coupled adenosine receptor signaling and lytic function of activated NK cells. *J. Immunol.* 175 4383–4391 10.4049/jimmunol.175.7.4383 16177079

[B124] Rech A. J. Mick R. Martin S. Recio A. Aqui N. A. Powell D. J. (2012). CD25 blockade depletes and selectively reprograms regulatory T cells in concert with immunotherapy in cancer patients. *Sci. Transl. Med.* 4:134ra62 10.1126/scitranslmed.3003330 PMC4425934 22593175

[B125] Rech A. J. Vonderheide R. H. (2009). Clinical use of anti-CD25 antibody daclizumab to enhance immune responses to tumor antigen vaccination by targeting regulatory T cells. *Ann. N. Y. Acad. Sci.* 1174 99–106 10.1111/j.1749-6632.2009.04939.x 19769742

[B126] Roncarolo M. G. Gregori S. Battaglia M. Bacchetta R. Fleischhauer K. Levings M. K. (2006). Interleukin-10-secreting type 1 regulatory T cells in rodents and humans. *Immunol. Rev.* 212 28–50 10.1111/j.0105-2896.2006.00420.x 16903904

[B127] Rts S. C. T. P. Agnandji S. T. Lell B. Fernandes J. F. Abossolo B. P. Methogo B. G. (2012). A phase 3 trial of RTS,S/AS01 malaria vaccine in African infants. *N. Engl. J. Med.* 367 2284–2295 10.1056/NEJMoa1208394 23136909 PMC10915853

[B128] Saison J. Ferry T. Demaret J. Maucort Boulch D. Venet F. Perpoint T. (2014). Association between discordant immunological response to highly active anti-retroviral therapy, regulatory T cell percentage, immune cell activation and very low-level viraemia in HIV-infected patients. *Clin. Exp. Immunol.* 176 401–409 10.1111/cei.12278 24460818 PMC4008985

[B129] Sakaguchi S. (2000). Regulatory T cells: key controllers of immunologic self-tolerance. *Cell* 101 455–458 10.1016/S0092-8674(00)80856-9 10850488

[B130] Sakaguchi S. Sakaguchi N. Asano M. Itoh M. Toda M. (1995). Immunologic self-tolerance maintained by activated T cells expressing IL-2 receptor alpha-chains (CD25). Breakdown of a single mechanism of self-tolerance causes various autoimmune diseases. *J. Immunol.* 155 1151–1164 7636184

[B131] Sallusto F. Lenig D. Forster R. Lipp M. Lanzavecchia A. (1999). Two subsets of memory T lymphocytes with distinct homing potentials and effector functions. *Nature* 401 708–712 10.1038/44385 10537110

[B132] Sansom D. M. Walker L. S. (2006). The role of CD28 and cytotoxic T-lymphocyte antigen-4 (CTLA-4) in regulatory T-cell biology. *Immunol. Rev.* 212 131–148 10.1111/j.0105-2896.2006.00419.x 16903911

[B133] Santner-Nanan B. Seddiki N. Zhu E. Quent V. Kelleher A. Fazekas De St Groth B. (2008). Accelerated age-dependent transition of human regulatory T cells to effector memory phenotype. *Int. Immunol.* 20 375–383 10.1093/intimm/dxm151 18195049

[B134] Scalzo-Inguanti K. Plebanski M. (2011). CD38 identifies a hypo-proliferative IL-13-secreting CD4^+^ T-cell subset that does not fit into existing naive and memory phenotype paradigms. *Eur. J. Immunol.* 41 1298–1308 10.1002/eji.201040726 21469087

[B135] Schmetterer K. G. Neunkirchner A. Pickl W. F. (2012). Naturally occurring regulatory T cells: markers, mechanisms, and manipulation. *FASEB J.* 26 2253–2276 10.1096/fj.11-193672 22362896

[B136] Scholzen A. Minigo G. Plebanski M. (2010). Heroes or villains? T regulatory cells in malaria infection. *Trends Parasitol.* 26 16–25 10.1016/j.pt.2009.10.004 19914134

[B137] Scholzen A. Mittag D. Rogerson S. J. Cooke B. M. Plebanski M. (2009). *Plasmodium falciparum*-mediated induction of human CD25Foxp3 CD4 T cells is independent of direct TCR stimulation and requires IL-2 IL-10 and TGFbeta. *PLoS Pathog.* 5:e1000543 10.1371/journal.ppat.1000543 PMC2718810 19680449

[B138] Seddiki N. Santner-Nanan B. Martinson J. Zaunders J. Sasson S. Landay A. (2006). Expression of interleukin (IL)-2 and IL-7 receptors discriminates between human regulatory and activated T cells. *J. Exp. Med.* 203 1693–1700 10.1084/jem.20060468 16818676 PMC2118333

[B139] Serana F. Chiarini M. Quiros-Roldan E. Gotti D. Zanotti C. Sottini A. (2014). Modulation of regulatory T-cell subsets in very long-term treated aviremic HIV(+) patients and untreated viremic patients. *Open AIDS J.* 8 1–6 10.2174/1874613601408010001 24627733 PMC3952203

[B140] Siegrist C. A. (2003). Mechanisms by which maternal antibodies influence infant vaccine responses: review of hypotheses and definition of main determinants. *Vaccine* 21 3406–3412 10.1016/S0264-410X(03)00342-6 12850349

[B141] Siegrist C. A. Barrios C. Martinez X. Brandt C. Berney M. Cordova M. (1998). Influence of maternal antibodies on vaccine responses: inhibition of antibody but not T cell responses allows successful early prime-boost strategies in mice. *Eur. J. Immunol.* 28 4138–4148 10.1002/(SICI)1521-4141(199812)28:12<4138::AID-IMMU4138>3.0.CO;2-L 9862350

[B142] Simone R. Zicca A. Saverino D. (2008). The frequency of regulatory CD3^+^CD8^+^CD28^-^ CD25^+^ T lymphocytes in human peripheral blood increases with age. *J. Leukoc. Biol.* 84 1454–1461 10.1189/jlb.0907627 18780874

[B143] Sitkovsky M. V. Ohta A. (2005). The ‘danger’ sensors that STOP the immune response: the A2 adenosine receptors? *Trends Immunol*. 26 299–304 10.1016/j.it.2005.04.004 15922945

[B144] Smith T. R. Kumar V. (2008). Revival of CD8^+^ Treg-mediated suppression. *Trends Immunol.* 29 337–342 10.1016/j.it.2008.04.002 18514574

[B145] Suzuki M. Jagger A. L. Konya C. Shimojima Y. Pryshchep S. Goronzy J. J. (2012). CD8^+^CD45RA^+^CCR7^+^FOXP3^+^ T cells with immunosuppressive properties: a novel subset of inducible human regulatory T cells. *J. Immunol.* 189 2118–2130 10.4049/jimmunol.1200122 22821963 PMC3424334

[B146] Suzuki M. Konya C. Goronzy J. J. Weyand C. M. (2008). Inhibitory CD8^+^ T cells in autoimmune disease. *Hum. Immunol.* 69 781–789 10.1016/j.humimm.2008.08.283 18812196 PMC2614126

[B147] Takahata Y. Nomura A. Takada H. Ohga S. Furuno K. Hikino S. (2004). CD25^+^CD4^+^ T cells in human cord blood: an immunoregulatory subset with naive phenotype and specific expression of forkhead box p3 (Foxp3) gene. *Exp. Hematol.* 32 622–629 10.1016/j.exphem.2004.03.012 15246158

[B148] Tan C. Reddy V. Dannull J. Ding E. Nair S. K. Tyler D. S. (2013). Impact of anti-CD25 monoclonal antibody on dendritic cell-tumor fusion vaccine efficacy in a murine melanoma model. *J. Transl. Med.* 11:148 10.1186/1479-5876-11-148 PMC3691646 23768240

[B149] Todryk S. M. Bejon P. Mwangi T. Plebanski M. Urban B. Marsh K. (2008). Correlation of memory T cell responses against TRAP with protection from clinical malaria, and CD4 CD25 high T cells with susceptibility in Kenyans. *PLoS ONE* 3:e2027 10.1371/journal.pone.0002027 PMC2323567 18446217

[B150] Tosello V. Odunsi K. Souleimanian N. E. Lele S. Shrikant P. Old L. J. (2008). Differential expression of CCR7 defines two distinct subsets of human memory CD4^+^CD25^+^ Tregs. *Clin. Immunol.* 126 291–302 10.1016/j.clim.2007.11.008 18166500

[B151] Uss E. Rowshani A. T. Hooibrink B. Lardy N. M. Van Lier R. A. Ten Berge I. J. (2006). CD103 is a marker for alloantigen-induced regulatory CD8^+^ T cells. *J. Immunol.* 177 2775–2783 10.4049/jimmunol.177.5.2775 16920912

[B152] Vieira P. L. Christensen J. R. Minaee S. O’Neill E. J. Barrat F. J. Boonstra A. (2004). IL-10-secreting regulatory T cells do not express Foxp3 but have comparable regulatory function to naturally occurring CD4^+^CD25^+^ regulatory T cells. *J. Immunol.* 172 5986–5993 10.4049/jimmunol.172.10.5986 15128781

[B153] Voo K. S. Bover L. Harline M. L. Vien L. T. Facchinetti V. Arima K. (2013). Antibodies targeting human OX40 expand effector T cells and block inducible and natural regulatory T cell function. *J. Immunol.* 191 3641–3650 10.4049/jimmunol.1202752 24014877 PMC3812678

[B154] Walker L. S. (2013). Treg and CTLA-4: two intertwining pathways to immune tolerance. *J. Autoimmun.* 45 49–57 10.1016/j.jaut.2013.06.006 23849743 PMC3989116

[B155] Walther B. Miles D. J. Waight P. Palmero M. S. Ojuola O. Touray E. S. (2012). Placental malaria is associated with attenuated CD4 T-cell responses to tuberculin PPD 12 months after BCG vaccination. *BMC Infect. Dis.* 12:6 10.1186/1471-2334-12-6 PMC3274427 22243970

[B156] Walther M. Jeffries D. Finney O. C. Njie M. Ebonyi A. Deininger S. (2009). Distinct roles for FOXP3 and FOXP3 CD4 T cells in regulating cellular immunity to uncomplicated and severe *Plasmodium falciparum* malaria. *PLoS Pathog.* 5:e1000364 10.1371/journal.ppat.1000364 PMC2658808 19343213

[B157] Walther M. Tongren J. E. Andrews L. Korbel D. King E. Fletcher H. (2005). Upregulation of TGF-beta, FOXP3 and CD4^+^CD25^+^ regulatory T cells correlates with more rapid parasite growth in human malaria infection. *Immunity* 23 287–296 10.1016/j.immuni.2005.08.006 16169501

[B158] Wammes L. J. Wiria A. E. Toenhake C. G. Hamid F. Liu K. Y. Suryani H. (2013). Asymptomatic plasmodial infection is associated with increased tumor necrosis factor receptor II-expressing regulatory T cells and suppressed type 2 immune responses. *J. Infect. Dis.* 207 1590–1599 10.1093/infdis/jit058 23408847

[B159] Wang W. H. Ming L. Wang Y. Kan Q. C. Zhang X. Y. (2013). High frequency of regulatory T cells among HIV type 1-infected men who have sex with men correlates with disease progression. *Chin. Med. J.* (*Engl.*) 126 2054–2061 23769557

[B160] Wing J. B. Sakaguchi S. (2012). Multiple Treg suppressive modules and their adaptability. *Front. Immunol.* 3:178 10.3389/fimmu.2012.00178 PMC3386489 22754556

[B161] Wing K. Ekmark A. Karlsson H. Rudin A. Suri-Payer E. (2002). Characterization of human CD25^+^ CD4^+^ T cells in thymus, cord and adult blood. *Immunology* 106 190–199 10.1046/j.1365-2567.2002.01412.x 12047748 PMC1782718

[B162] Wing K. Larsson P. Sandstrom K. Lundin S. B. Suri-Payer E. Rudin A. (2005). CD4^+^ CD25^+^ FOXP3^+^ regulatory T cells from human thymus and cord blood suppress antigen-specific T cell responses. *Immunology* 115 516–525 10.1111/j.1365-2567.2005.02186.x 16011520 PMC1782183

[B163] Wing K. Yamaguchi T. Sakaguchi S. (2011). Cell-autonomous and -non-autonomous roles of CTLA-4 in immune regulation. *Trends Immunol.* 32 428–433 10.1016/j.it.2011.06.002 21723783

[B164] Wu J. J. Chen G. Liu J. Wang T. Zheng W. Cao Y. M. (2010). Natural regulatory T cells mediate the development of cerebral malaria by modifying the pro-inflammatory response. *Parasitol. Int.* 59 232–241 10.1016/j.parint.2010.02.007 20219695

[B165] Yeung L. T. King S. M. Roberts E. A. (2001). Mother-to-infant transmission of hepatitis C virus. *Hepatology* 34 223–229 10.1053/jhep.2001.25885 11481604

[B166] Zandvoort A. Timens W. (2002). The dual function of the splenic marginal zone: essential for initiation of anti-TI-2 responses but also vital in the general first-line defense against blood-borne antigens. *Clin. Exp. Immunol.* 130 4–11 10.1046/j.1365-2249.2002.01953.x 12296846 PMC1906503

